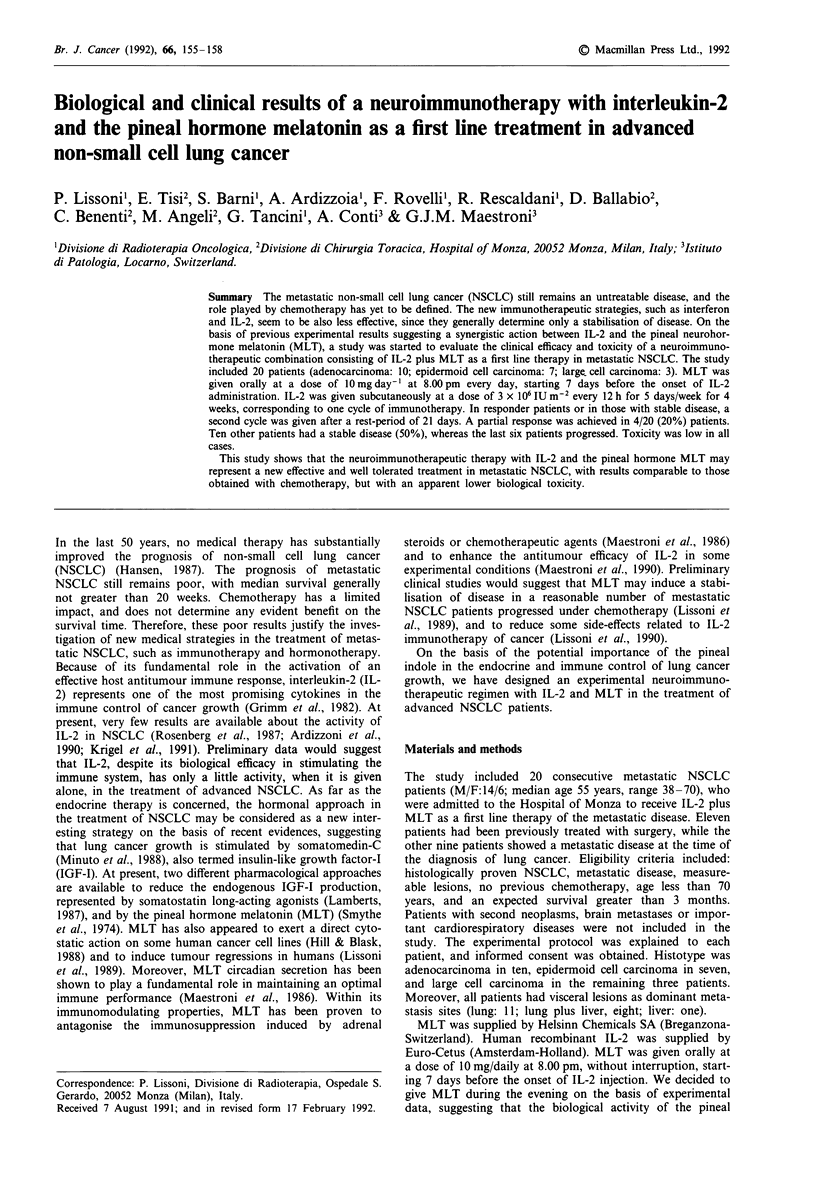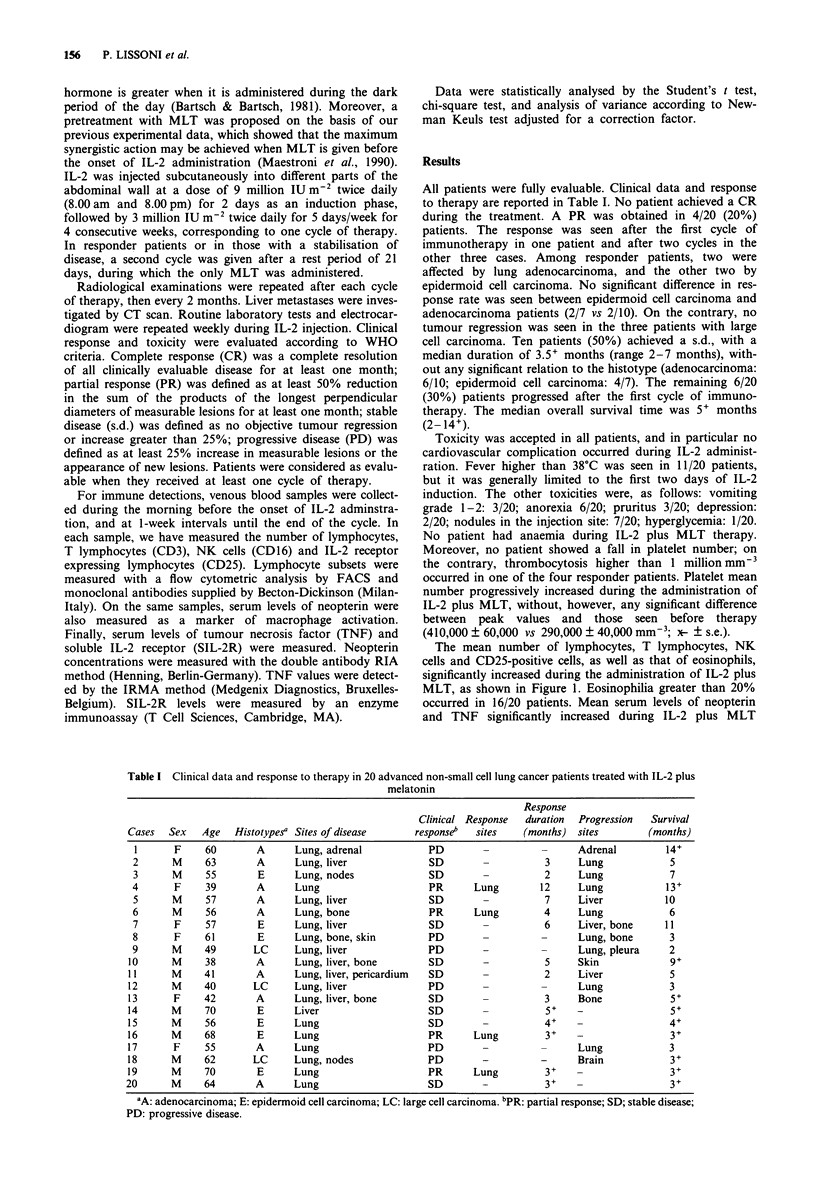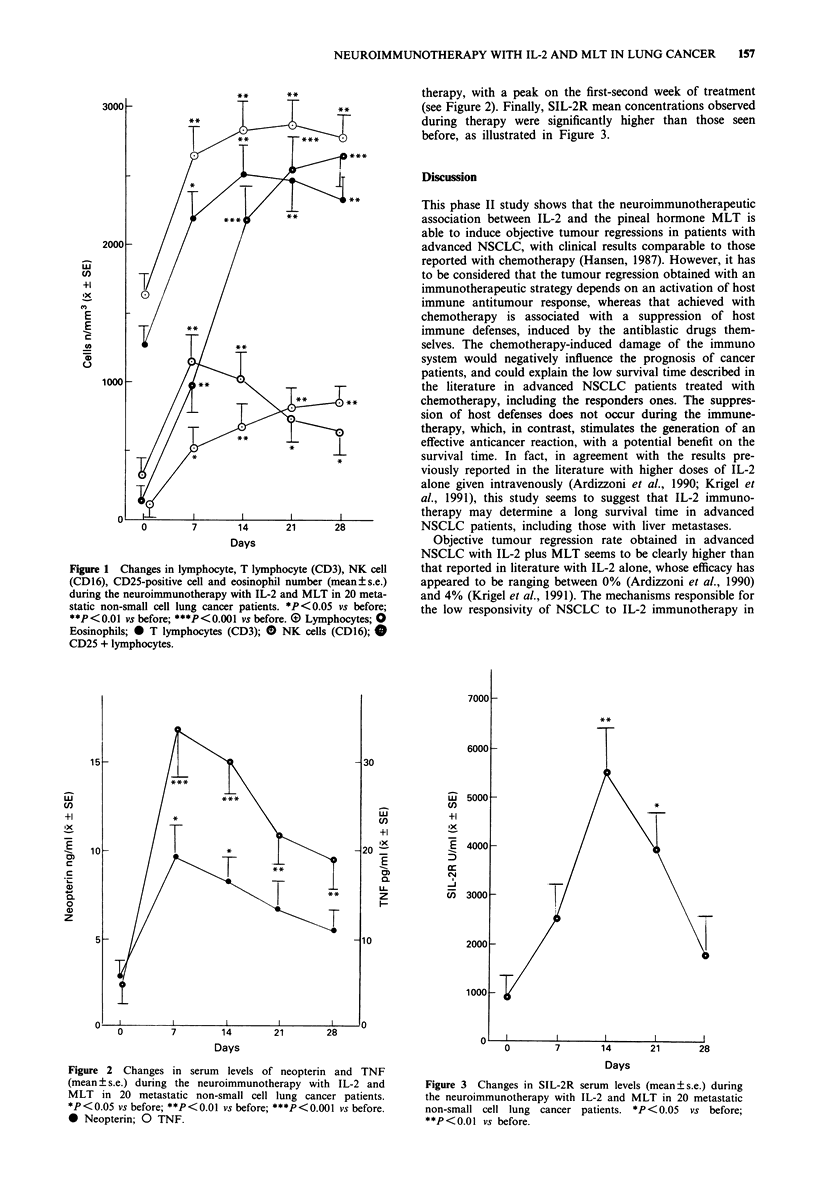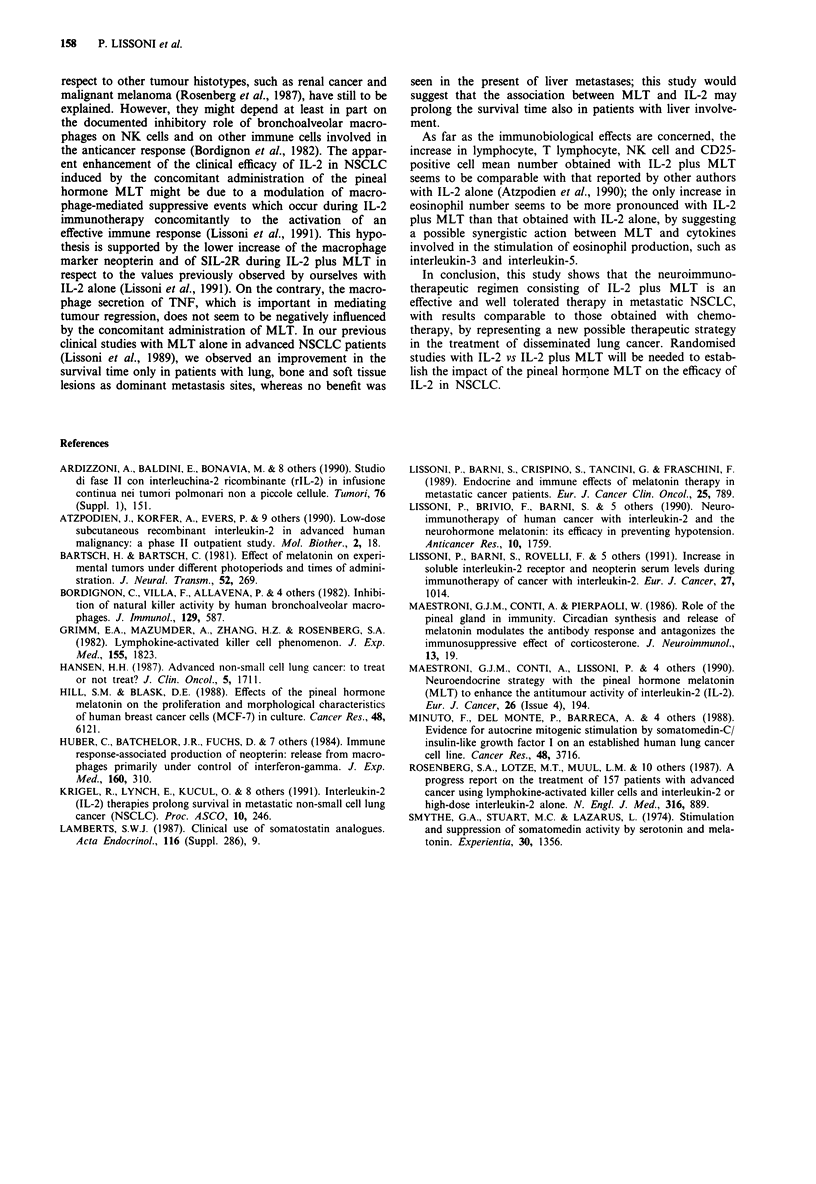# Biological and clinical results of a neuroimmunotherapy with interleukin-2 and the pineal hormone melatonin as a first line treatment in advanced non-small cell lung cancer.

**DOI:** 10.1038/bjc.1992.234

**Published:** 1992-07

**Authors:** P. Lissoni, E. Tisi, S. Barni, A. Ardizzoia, F. Rovelli, R. Rescaldani, D. Ballabio, C. Benenti, M. Angeli, G. Tancini

**Affiliations:** Divisione di Radioterapia Oncologica, Hospital of Monza, Milan, Italy.

## Abstract

The metastatic non-small cell lung cancer (NSCLC) still remains an untreatable disease, and the role played by chemotherapy has yet to be defined. The new immunotherapeutic strategies, such as interferon and IL-2, seem to be also less effective, since they generally determine only a stabilisation of disease. On the basis of previous experimental results suggesting a synergistic action between IL-2 and the pineal neurohormone melatonin (MLT), a study was started to evaluate the clinical efficacy and toxicity of a neuroimmunotherapeutic combination consisting of IL-2 plus MLT as a first line therapy in metastatic NSCLC. The study included 20 patients (adenocarcinoma: 10; epidermoid cell carcinoma: 7; large cell carcinoma: 3). MLT was given orally at a dose of 10 mg day-1 at 8.00 pm every day, starting 7 days before the onset of IL-2 administration. IL-2 was given subcutaneously at a dose of 3 x 10(6) IU m-2 every 12 h for 5 days/week for 4 weeks, corresponding to one cycle of immunotherapy. In responder patients or in those with stable disease, a second cycle was given after a rest-period of 21 days. A partial response was achieved in 4/20 (20%) patients. Ten other patients had a stable disease (50%), whereas the last six patients progressed. Toxicity was low in all cases. This study shows that the neuroimmunotherapeutic therapy with IL-2 and the pineal hormone MLT may represent a new effective and well tolerated treatment in metastatic NSCLC, with results comparable to those obtained with chemotherapy, but with an apparent lower biological toxicity.


Br. J. Cancer (1992), 66, 155-158 © Macmillan Press Ltd., 1992

### Biological and clinical results of a neuroimmunotherapy with interleukin-2 and the pineal hormone melatonin as a first line treatment in advanced non-small cell lung cancer

P. Lissoni[1], E. Tisi[2], S. Barni[1], A. Ardizzoia[1], F. Rovelli[1], R. Rescaldani[1], D. Ballabio[2], C. Benenti[2], M. Angeli[2], G. Tancini[1], A. Conti[3] & G.J.M. Maestroni[3]

[1]*Divisione di Radioterapia Oncologica,* [2]*Divisione di Chirurgia Toracica, Hospital of Monza, 20052 Monza, Milan, Italy;* [3]*Istituto di Patologia, Locarno, Switzerland.*

**Summary** The metastatic non-small cell lung cancer (NSCLC) still remains an untreatable disease, and the role played by chemotherapy has yet to be defined. The new immunotherapeutic strategies, such as interferon and IL-2, seem to be also less effective, since they generally determine only a stabilisation of disease. On the basis of previous experimental results suggesting a synergistic action between IL-2 and the pineal neurohormone melatonin (MLT), a study was started to evaluate the clinical efficacy and toxicity of a neuroimmunotherapeutic combination consisting of IL-2 plus MLT as a first line therapy in metastatic NSCLC. The study included 20 patients (adenocarcinoma: 10; epidermoid cell carcinoma: 7; large cell carcinoma: 3). MLT was given orally at a dose of 10 mg $day^{-1}$ at 8.00 pm every day, starting 7 days before the onset of IL-2 administration. IL-2 was given subcutaneously at a dose of $3 \times 10^6$ IU $m^{-2}$ every 12 h for 5 days/week for 4 weeks, corresponding to one cycle of immunotherapy. In responder patients or in those with stable disease, a second cycle was given after a rest-period of 21 days. A partial response was achieved in 4/20 (20%) patients. Ten other patients had a stable disease (50%), whereas the last six patients progressed. Toxicity was low in all cases.

This study shows that the neuroimmunotherapeutic therapy with IL-2 and the pineal hormone MLT may represent a new effective and well tolerated treatment in metastatic NSCLC, with results comparable to those obtained with chemotherapy, but with an apparent lower biological toxicity.

In the last 50 years, no medical therapy has substantially improved the prognosis of non-small cell lung cancer (NSCLC) (Hansen, 1987). The prognosis of metastatic NSCLC still remains poor, with median survival generally not greater than 20 weeks. Chemotherapy has a limited impact, and does not determine any evident benefit on the survival time. Therefore, these poor results justify the investigation of new medical strategies in the treatment of metastatic NSCLC, such as immunotherapy and hormonotherapy. Because of its fundamental role in the activation of an effective host antitumour immune response, interleukin-2 (IL-2) represents one of the most promising cytokines in the immune control of cancer growth (Grimm *et al.*, 1982). At present, very few results are available about the activity of IL-2 in NSCLC (Rosenberg *et al.*, 1987; Ardizzoni *et al.*, 1990; Krigel *et al.*, 1991). Preliminary data would suggest that IL-2, despite its biological efficacy in stimulating the immune system, has only a little activity, when it is given alone, in the treatment of advanced NSCLC. As far as the endocrine therapy is concerned, the hormonal approach in the treatment of NSCLC may be considered as a new interesting strategy on the basis of recent evidences, suggesting that lung cancer growth is stimulated by somatomedin-C (Minuto *et al.*, 1988), also termed insulin-like growth factor-I (IGF-I). At present, two different pharmacological approaches are available to reduce the endogenous IGF-I production, represented by somatostatin long-acting agonists (Lamberts, 1987), and by the pineal hormone melatonin (MLT) (Smythe *et al.*, 1974). MLT has also appeared to exert a direct cytostatic action on some human cancer cell lines (Hill & Blask, 1988) and to induce tumour regressions in humans (Lissoni *et al.*, 1989). Moreover, MLT circadian secretion has been shown to play a fundamental role in maintaining an optimal immune performance (Maestroni *et al.*, 1986). Within its immunomodulating properties, MLT has been proven to antagonise the immunosuppression induced by adrenal steroids or chemotherapeutic agents (Maestroni *et al.*, 1986) and to enhance the antitumour efficacy of IL-2 in some experimental conditions (Maestroni *et al.*, 1990). Preliminary clinical studies would suggest that MLT may induce a stabilisation of disease in a reasonable number of mestastatic NSCLC patients progressed under chemotherapy (Lissoni *et al.*, 1989), and to reduce some side-effects related to IL-2 immunotherapy of cancer (Lissoni *et al.*, 1990).

On the basis of the potential importance of the pineal indole in the endocrine and immune control of lung cancer growth, we have designed an experimental neuroimmunotherapeutic regimen with IL-2 and MLT in the treatment of advanced NSCLC patients.

### Materials and methods

The study included 20 consecutive metastatic NSCLC patients (M/F:14/6; median age 55 years, range 38-70), who were admitted to the Hospital of Monza to receive IL-2 plus MLT as a first line therapy of the metastatic disease. Eleven patients had been previously treated with surgery, while the other nine patients showed a metastatic disease at the time of the diagnosis of lung cancer. Eligibility criteria included: histologically proven NSCLC, metastatic disease, measureable lesions, no previous chemotherapy, age less than 70 years, and an expected survival greater than 3 months. Patients with second neoplasms, brain metastases or important cardiorespiratory diseases were not included in the study. The experimental protocol was explained to each patient, and informed consent was obtained. Histotype was adenocarcinoma in ten, epidermoid cell carcinoma in seven, and large cell carcinoma in the remaining three patients. Moreover, all patients had visceral lesions as dominant metastasis sites (lung: 11; lung plus liver, eight; liver: one).

MLT was supplied by Helsinn Chemicals SA (Breganzona-Switzerland). Human recombinant IL-2 was supplied by Euro-Cetus (Amsterdam-Holland). MLT was given orally at a dose of 10 mg/daily at 8.00 pm, without interruption, starting 7 days before the onset of IL-2 injection. We decided to give MLT during the evening on the basis of experimental data, suggesting that the biological activity of the pineal

Correspondence: P. Lissoni, Divisione di Radioterapia, Ospedale S. Gerardo, 20052 Monza (Milan), Italy.
Received 7 August 1991; and in revised form 17 February 1992.

hormone is greater when it is administered during the dark period of the day (Bartsch & Bartsch, 1981). Moreover, a pretreatment with MLT was proposed on the basis of our previous experimental data, which showed that the maximum synergistic action may be achieved when MLT is given before the onset of IL-2 administration (Maestroni et al., 1990). IL-2 was injected subcutaneously into different parts of the abdominal wall at a dose of 9 million IU $m^{-2}$ twice daily (8.00 am and 8.00 pm) for 2 days as an induction phase, followed by 3 million IU $m^{-2}$ twice daily for 5 days/week for 4 consecutive weeks, corresponding to one cycle of therapy. In responder patients or in those with a stabilisation of disease, a second cycle was given after a rest period of 21 days, during which the only MLT was administered.

Radiological examinations were repeated after each cycle of therapy, then every 2 months. Liver metastases were investigated by CT scan. Routine laboratory tests and electrocardiogram were repeated weekly during IL-2 injection. Clinical response and toxicity were evaluated according to WHO criteria. Complete response (CR) was a complete resolution of all clinically evaluable disease for at least one month; partial response (PR) was defined as at least 50% reduction in the sum of the products of the longest perpendicular diameters of measurable lesions for at least one month; stable disease (s.d.) was defined as no objective tumour regression or increase greater than 25%; progressive disease (PD) was defined as at least 25% increase in measurable lesions or the appearance of new lesions. Patients were considered as evaluable when they received at least one cycle of therapy.

For immune detections, venous blood samples were collected during the morning before the onset of IL-2 adminstration, and at 1-week intervals until the end of the cycle. In each sample, we have measured the number of lymphocytes, T lymphocytes (CD3), NK cells (CD16) and IL-2 receptor expressing lymphocytes (CD25). Lymphocyte subsets were measured with a flow cytometric analysis by FACS and monoclonal antibodies supplied by Becton-Dickinson (Milan-Italy). On the same samples, serum levels of neopterin were also measured as a marker of macrophage activation. Finally, serum levels of tumour necrosis factor (TNF) and soluble IL-2 receptor (SIL-2R) were measured. Neopterin concentrations were measured with the double antibody RIA method (Henning, Berlin-Germany). TNF values were detected by the IRMA method (Medgenix Diagnostics, Bruxelles-Belgium). SIL-2R levels were measured by an enzyme immunoassay (T Cell Sciences, Cambridge, MA).

Data were statistically analysed by the Student's $t$ test, chi-square test, and analysis of variance according to Newman Keuls test adjusted for a correction factor.

### Results

All patients were fully evaluable. Clinical data and response to therapy are reported in Table I. No patient achieved a CR during the treatment. A PR was obtained in 4/20 (20%) patients. The response was seen after the first cycle of immunotherapy in one patient and after two cycles in the other three cases. Among responder patients, two were affected by lung adenocarcinoma, and the other two by epidermoid cell carcinoma. No significant difference in response rate was seen between epidermoid cell carcinoma and adenocarcinoma patients (2/7 vs 2/10). On the contrary, no tumour regression was seen in the three patients with large cell carcinoma. Ten patients (50%) achieved a s.d., with a median duration of $3.5^+$ months (range 2–7 months), without any significant relation to the histotype (adenocarcinoma: 6/10; epidermoid cell carcinoma: 4/7). The remaining 6/20 (30%) patients progressed after the first cycle of immunotherapy. The median overall survival time was $5^+$ months ($2-14^+$).

Toxicity was accepted in all patients, and in particular no cardiovascular complication occurred during IL-2 administration. Fever higher than 38°C was seen in 11/20 patients, but it was generally limited to the first two days of IL-2 induction. The other toxicities were, as follows: vomiting grade 1–2: 3/20; anorexia 6/20; pruritus 3/20; depression: 2/20; nodules in the injection site: 7/20; hyperglycemia: 1/20. No patient had anaemia during IL-2 plus MLT therapy. Moreover, no patient showed a fall in platelet number; on the contrary, thrombocytosis higher than 1 million $mm^{-3}$ occurred in one of the four responder patients. Platelet mean number progressively increased during the administration of IL-2 plus MLT, without, however, any significant difference between peak values and those seen before therapy (410,000 ± 60,000 vs 290,000 ± 40,000 $mm^{-3}$; $\bar{x}$ ± s.e.).

The mean number of lymphocytes, T lymphocytes, NK cells and CD25-positive cells, as well as that of eosinophils, significantly increased during the administration of IL-2 plus MLT, as shown in Figure 1. Eosinophilia greater than 20% occurred in 16/20 patients. Mean serum levels of neopterin and TNF significantly increased during IL-2 plus MLT

**Table I** Clinical data and response to therapy in 20 advanced non-small cell lung cancer patients treated with IL-2 plus melatonin

| *Cases* | *Sex* | *Age* | *Histotypes*[a] | *Sites of disease* | *Clinical response*[b] | *Response sites* | *Response duration (months)* | *Progression sites* | *Survival (months)* |
|---|---|---|---|---|---|---|---|---|---|
| 1 | F | 60 | A | Lung, adrenal | PD | – | – | Adrenal | $14^+$ |
| 2 | M | 63 | A | Lung, liver | SD | – | 3 | Lung | 5 |
| 3 | M | 55 | E | Lung, nodes | SD | – | 2 | Lung | 7 |
| 4 | F | 39 | A | Lung | PR | Lung | 12 | Lung | $13^+$ |
| 5 | M | 57 | A | Lung, liver | SD | – | 7 | Liver | 10 |
| 6 | M | 56 | A | Lung, bone | PR | Lung | 4 | Lung | 6 |
| 7 | F | 57 | E | Lung, liver | SD | – | 6 | Liver, bone | 11 |
| 8 | F | 61 | E | Lung, bone, skin | PD | – | – | Lung, bone | 3 |
| 9 | M | 49 | LC | Lung, liver | PD | – | – | Lung, pleura | 2 |
| 10 | M | 38 | A | Lung, liver, bone | SD | – | 5 | Skin | $9^+$ |
| 11 | M | 41 | A | Lung, liver, pericardium | SD | – | 2 | Liver | 5 |
| 12 | M | 40 | LC | Lung, liver | PD | – | – | Lung | 3 |
| 13 | F | 42 | A | Lung, liver, bone | SD | – | 3 | Bone | $5^+$ |
| 14 | M | 70 | E | Liver | SD | – | $5^+$ | – | $5^+$ |
| 15 | M | 56 | E | Lung | SD | – | $4^+$ | – | $4^+$ |
| 16 | M | 68 | E | Lung | PR | Lung | $3^+$ | – | $3^+$ |
| 17 | F | 55 | A | Lung | PD | – | – | Lung | 3 |
| 18 | M | 62 | LC | Lung, nodes | PD | – | – | Brain | $3^+$ |
| 19 | M | 70 | E | Lung | PR | Lung | $3^+$ | – | $3^+$ |
| 20 | M | 64 | A | Lung | SD | – | $3^+$ | – | $3^+$ |

[a]A: adenocarcinoma; E: epidermoid cell carcinoma; LC: large cell carcinoma. [b]PR: partial response; SD; stable disease; PD: progressive disease.

**Figure 1** Changes in lymphocyte, T lymphocyte (CD3), NK cell (CD16), CD25-positive cell and eosinophil number (mean ± s.e.) during the neuroimmunotherapy with IL-2 and MLT in 20 metastatic non-small cell lung cancer patients. *$P<0.05$ *vs* before; **$P<0.01$ *vs* before; ***$P<0.001$ *vs* before. ⊕ Lymphocytes; O Eosinophils; ● T lymphocytes (CD3); ⊖ NK cells (CD16); ⊗ CD25 + lymphocytes.

therapy, with a peak on the first-second week of treatment (see Figure 2). Finally, SIL-2R mean concentrations observed during therapy were significantly higher than those seen before, as illustrated in Figure 3.

### Discussion

This phase II study shows that the neuroimmunotherapeutic association between IL-2 and the pineal hormone MLT is able to induce objective tumour regressions in patients with advanced NSCLC, with clinical results comparable to those reported with chemotherapy (Hansen, 1987). However, it has to be considered that the tumour regression obtained with an immunotherapeutic strategy depends on an activation of host immune antitumour response, whereas that achieved with chemotherapy is associated with a suppression of host immune defenses, induced by the antiblastic drugs themselves. The chemotherapy-induced damage of the immuno system would negatively influence the prognosis of cancer patients, and could explain the low survival time described in the literature in advanced NSCLC patients treated with chemotherapy, including the responders ones. The suppression of host defenses does not occur during the immune-therapy, which, in contrast, stimulates the generation of an effective anticancer reaction, with a potential benefit on the survival time. In fact, in agreement with the results previously reported in the literature with higher doses of IL-2 alone given intravenously (Ardizzoni *et al.*, 1990; Krigel *et al.*, 1991), this study seems to suggest that IL-2 immunotherapy may determine a long survival time in advanced NSCLC patients, including those with liver metastases.

Objective tumour regression rate obtained in advanced NSCLC with IL-2 plus MLT seems to be clearly higher than that reported in literature with IL-2 alone, whose efficacy has appeared to be ranging between 0% (Ardizzoni *et al.*, 1990) and 4% (Krigel *et al.*, 1991). The mechanisms responsible for the low responsivity of NSCLC to IL-2 immunotherapy in

**Figure 2** Changes in serum levels of neopterin and TNF (mean ± s.e.) during the neuroimmunotherapy with IL-2 and MLT in 20 metastatic non-small cell lung cancer patients. *$P<0.05$ *vs* before; **$P<0.01$ *vs* before; ***$P<0.001$ *vs* before. ● Neopterin; O TNF.

**Figure 3** Changes in SIL-2R serum levels (mean ± s.e.) during the neuroimmunotherapy with IL-2 and MLT in 20 metastatic non-small cell lung cancer patients. *$P<0.05$ *vs* before; **$P<0.01$ *vs* before.

respect to other tumour histotypes, such as renal cancer and malignant melanoma (Rosenberg *et al.*, 1987), have still to be explained. However, they might depend at least in part on the documented inhibitory role of bronchoalveolar macrophages on NK cells and on other immune cells involved in the anticancer response (Bordignon *et al.*, 1982). The apparent enhancement of the clinical efficacy of IL-2 in NSCLC induced by the concomitant administration of the pineal hormone MLT might be due to a modulation of macrophage-mediated suppressive events which occur during IL-2 immunotherapy concomitantly to the activation of an effective immune response (Lissoni *et al.*, 1991). This hypothesis is supported by the lower increase of the macrophage marker neopterin and of SIL-2R during IL-2 plus MLT in respect to the values previously observed by ourselves with IL-2 alone (Lissoni *et al.*, 1991). On the contrary, the macrophage secretion of TNF, which is important in mediating tumour regression, does not seem to be negatively influenced by the concomitant administration of MLT. In our previous clinical studies with MLT alone in advanced NSCLC patients (Lissoni *et al.*, 1989), we observed an improvement in the survival time only in patients with lung, bone and soft tissue lesions as dominant metastasis sites, whereas no benefit was seen in the present of liver metastases; this study would suggest that the association between MLT and IL-2 may prolong the survival time also in patients with liver involvement.

As far as the immunobiological effects are concerned, the increase in lymphocyte, T lymphocyte, NK cell and CD25-positive cell mean number obtained with IL-2 plus MLT seems to be comparable with that reported by other authors with IL-2 alone (Atzpodien *et al.*, 1990); the only increase in eosinophil number seems to be more pronounced with IL-2 plus MLT than that obtained with IL-2 alone, by suggesting a possible synergistic action between MLT and cytokines involved in the stimulation of eosinophil production, such as interleukin-3 and interleukin-5.

In conclusion, this study shows that the neuroimmunotherapeutic regimen consisting of IL-2 plus MLT is an effective and well tolerated therapy in metastatic NSCLC, with results comparable to those obtained with chemotherapy, by representing a new possible therapeutic strategy in the treatment of disseminated lung cancer. Randomised studies with IL-2 *vs* IL-2 plus MLT will be needed to establish the impact of the pineal hormone MLT on the efficacy of IL-2 in NSCLC.

### References

ARDIZZONI, A., BALDINI, E., BONAVIA, M. & 8 others (1990). Studio di fase II con interleuchina-2 ricombinante (rIL-2) in infusione continua nei tumori polmonari non a piccole cellule. *Tumori*, **76** (Suppl. 1), 151.

ATZPODIEN, J., KORFER, A., EVERS, P. & 9 others (1990). Low-dose subcutaneous recombinant interleukin-2 in advanced human malignancy: a phase II outpatient study. *Mol. Biother.*, **2**, 18.

BARTSCH, H. & BARTSCH, C. (1981). Effect of melatonin on experimental tumors under different photoperiods and times of administration. *J. Neural. Transm.*, **52**, 269.

BORDIGNON, C., VILLA, F., ALLAVENA, P. & 4 others (1982). Inhibition of natural killer activity by human bronchoalveolar macrophages. *J. Immunol.*, **129**, 587.

GRIMM, E.A., MAZUMDER, A., ZHANG, H.Z. & ROSENBERG, S.A. (1982). Lymphokine-activated killer cell phenomenon. *J. Exp. Med.*, **155**, 1823.

HANSEN, H.H. (1987). Advanced non-small cell lung cancer: to treat or not treat? *J. Clin. Oncol.*, **5**, 1711.

HILL, S.M. & BLASK, D.E. (1988). Effects of the pineal hormone melatonin on the proliferation and morphological characteristics of human breast cancer cells (MCF-7) in culture. *Cancer Res.*, **48**, 6121.

HUBER, C., BATCHELOR, J.R., FUCHS, D. & 7 others (1984). Immune response-associated production of neopterin: release from macrophages primarily under control of interferon-gamma. *J. Exp. Med.*, **160**, 310.

KRIGEL, R., LYNCH, E., KUCUL, O. & 8 others (1991). Interleukin-2 (IL-2) therapies prolong survival in metastatic non-small cell lung cancer (NSCLC). *Proc. ASCO*, **10**, 246.

LAMBERTS, S.W.J. (1987). Clinical use of somatostatin analogues. *Acta Endocrinol.*, **116** (Suppl. 286), 9.

LISSONI, P., BARNI, S., CRISPINO, S., TANCINI, G. & FRASCHINI, F. (1989). Endocrine and immune effects of melatonin therapy in metastatic cancer patients. *Eur. J. Cancer Clin. Oncol.*, **25**, 789.

LISSONI, P., BRIVIO, F., BARNI, S. & 5 others (1990). Neuroimmunotherapy of human cancer with interleukin-2 and the neurohormone melatonin: its efficacy in preventing hypotension. *Anticancer Res.*, **10**, 1759.

LISSONI, P., BARNI, S., ROVELLI, F. & 5 others (1991). Increase in soluble interleukin-2 receptor and neopterin serum levels during immunotherapy of cancer with interleukin-2. *Eur. J. Cancer*, **27**, 1014.

MAESTRONI, G.J.M., CONTI, A. & PIERPAOLI, W. (1986). Role of the pineal gland in immunity. Circadian synthesis and release of melatonin modulates the antibody response and antagonizes the immunosuppressive effect of corticosterone. *J. Neuroimmunol.*, **13**, 19.

MAESTRONI, G.J.M., CONTI, A., LISSONI, P. & 4 others (1990). Neuroendocrine strategy with the pineal hormone melatonin (MLT) to enhance the antitumour activity of interleukin-2 (IL-2). *Eur. J. Cancer*, **26** (Issue 4), 194.

MINUTO, F., DEL MONTE, P., BARRECA, A. & 4 others (1988). Evidence for autocrine mitogenic stimulation by somatomedin-C/insulin-like growth factor I on an established human lung cancer cell line. *Cancer Res.*, **48**, 3716.

ROSENBERG, S.A., LOTZE, M.T., MUUL, L.M. & 10 others (1987). A progress report on the treatment of 157 patients with advanced cancer using lymphokine-activated killer cells and interleukin-2 or high-dose interleukin-2 alone. *N. Engl. J. Med.*, **316**, 889.

SMYTHE, G.A., STUART, M.C. & LAZARUS, L. (1974). Stimulation and suppression of somatomedin activity by serotonin and melatonin. *Experientia*, **30**, 1356.